# Rehabilitation of severely injured patients

**DOI:** 10.1007/s00068-025-02830-w

**Published:** 2025-03-25

**Authors:** Thomas Mendel, Ingo Marzi, Christiane Anke

**Affiliations:** 1 Clinic for Physical and Rehabilitative Medicine BG Klinikum Bergmannstrost Halle, Halle, Germany; 2 Department of Trauma Surgery and Orthopedics, University Hospital, Goethe University Frankfurt, Frankfurt Am Main, Germany; 3 Department of Trauma and Reconstructive Surgery, BG Klinikum Bergmannstrost, Halle, Germany

**Keywords:** Trauma, Rehabilitation, Outcome, Trauma care

## Abstract

Rehabilitation is a vital component of the holistic care of severely injured patients, addressing physical limitations, preventing complications, and promoting social and professional reintegration. Tailored measures are required across all phases of care, depending on individual injury patterns. Adequate early rehabilitation within inpatient settings necessitates appropriate personnel and infrastructure. Rehabilitation teams must include specialists from diverse therapeutic disciplines. Although financial frameworks vary by country, comprehensive funding for high-quality therapy programmes is essential for effective treatment.

## Introduction

Rehabilitation plays an integral role in the care of severely injured patients, bridging the gap between acute medical treatment and professional or social reintegration. This encompasses measures to eliminate, reduce, or compensate for physical limitations caused by trauma, mitigate complications, and address any resulting care needs. Rehabilitation efforts should commence during acute inpatient treatment and continue throughout all phases of polytrauma care, as they significantly influence the extent of a patient’s recovery and long-term outcomes.

The type and scope of rehabilitative measures depend on the injury pattern and severity. Broadly, therapeutic strategies can be broadly categorised into three primary domains:

1. Injuries to the head and brain.
2. Injuries to the trunk including thoracic, abdominal and intrapelvic organ systems.
3. Injuries to the extremities.

**Fig. 1:**
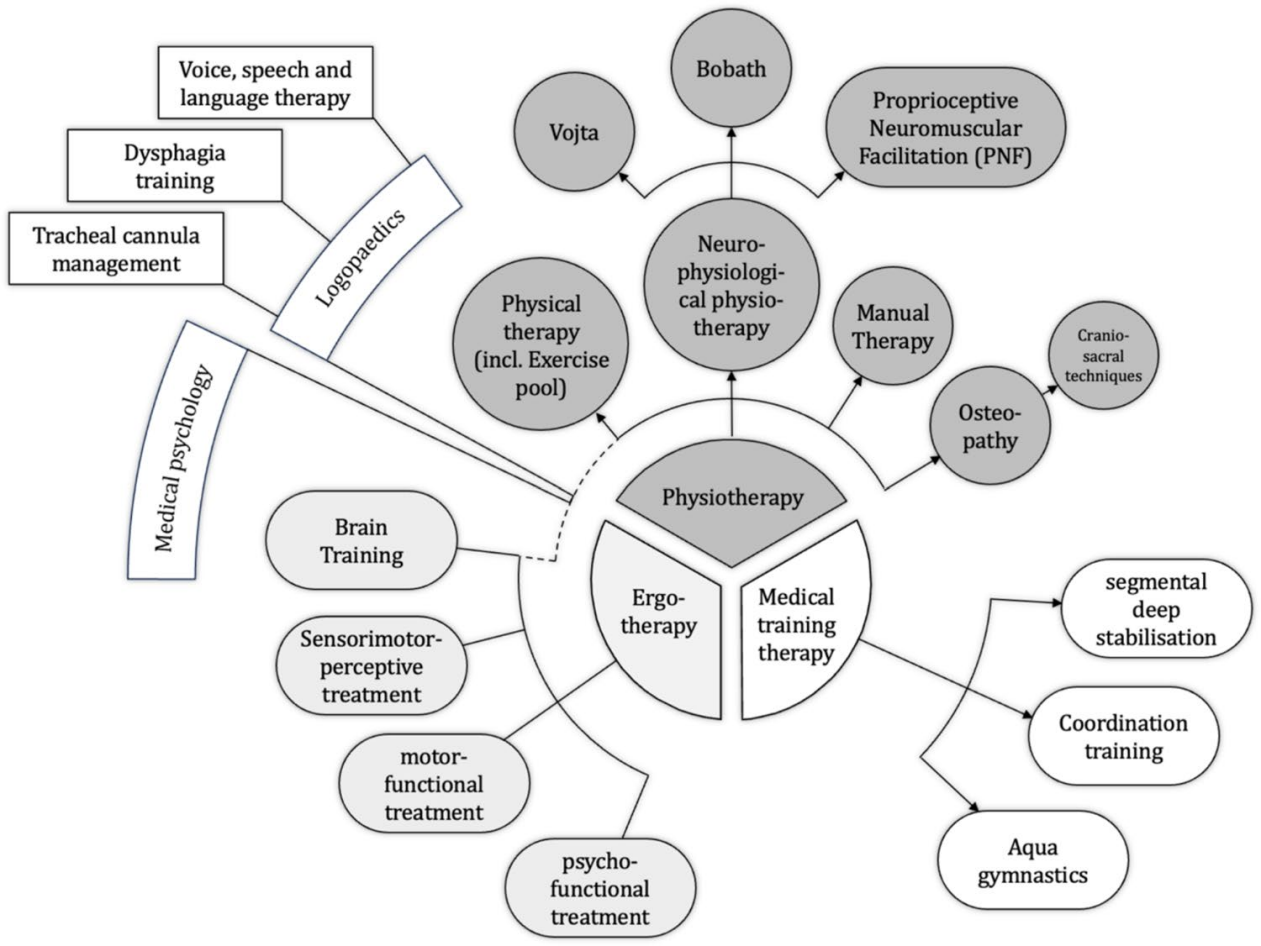
Treatment approach to severely injured patients with traumatic brain injury and/or spinal trauma

**Fig. 2:**
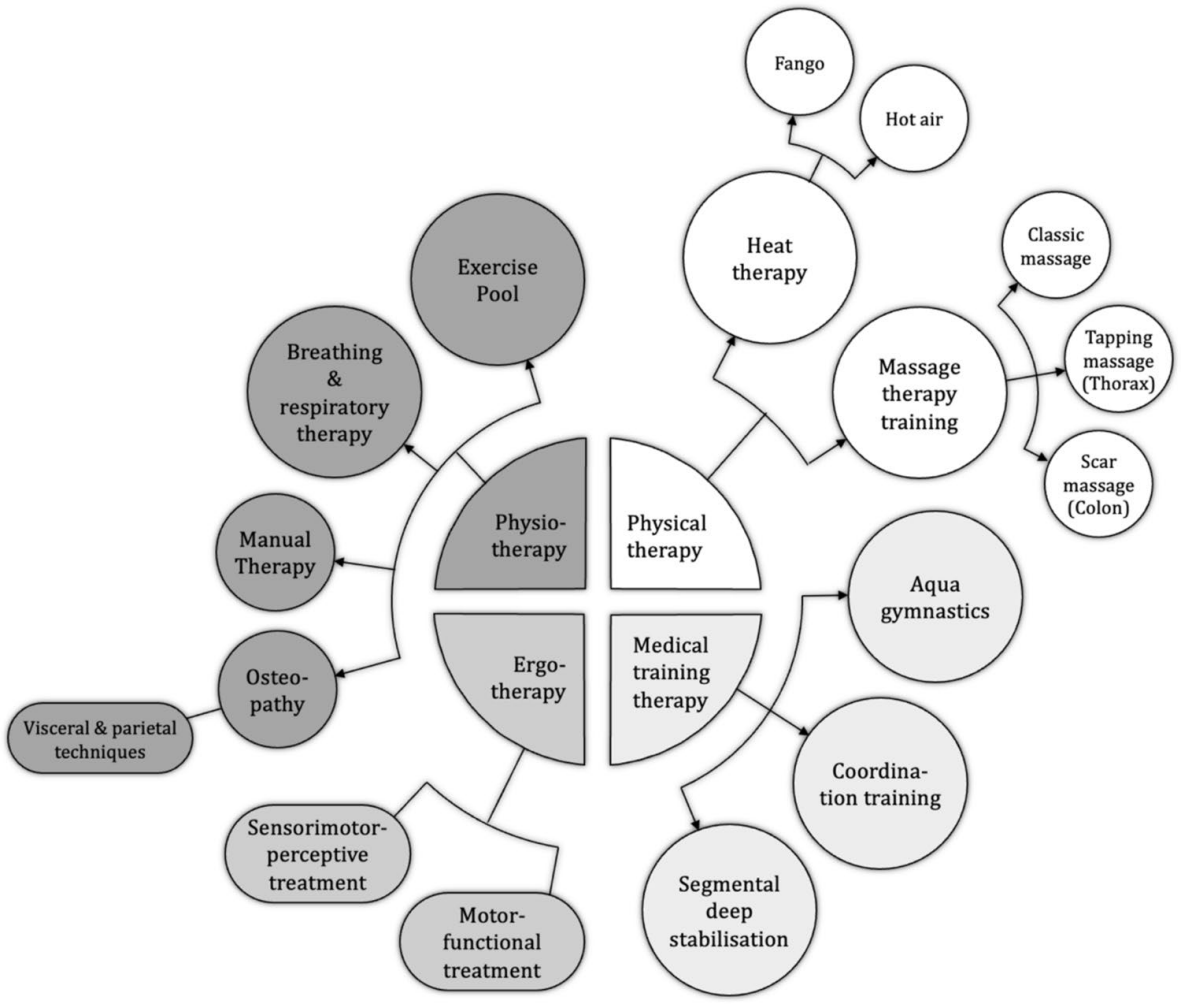
Treatment approach to severely injured persons with body trunk injuries

**Fig. 3:**
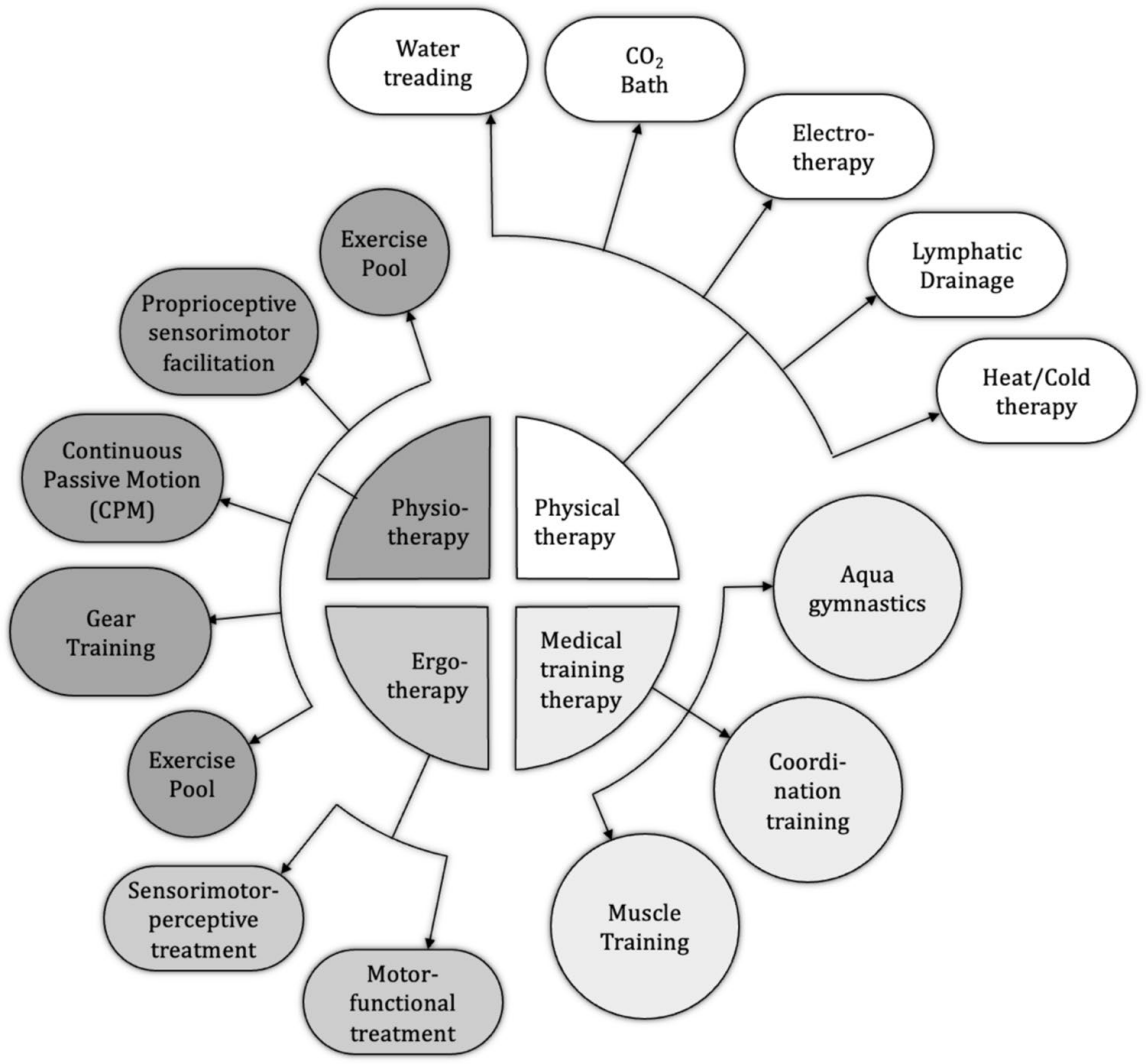
Treatment approach to severely injured patients with extremity injuries

Here, the rehabilitation goals focus in varying degrees on cognitive, motor and sensory deficits along with general thromboembolism prophylaxis in the context of limited mobility due to injuries (Figs. 1, 2 and 3).

## Inpatient rehabilitation/physiotherapy

### Personnel requirements

#### Medical management

Rehabilitation of severely injured patients requires medical oversight by specialists in orthopaedics and trauma surgery or general surgery with trauma subspecialisation. Alternatively, specialists in physical and rehabilitative medicine can fulfil this role. These professionals should also have socio-medical competence through additional training in rehabilitation or social medicine. Accreditation for further training in these qualifications is recommended.

The multidisciplinary rehabilitation team should include the following disciplines:

- Healthcare and nursing
- Physiotherapy
- Massage therapy
- Ergotherapy
- Sports therapy
- Clinical psychology and neuropsychology
- Psychotherapy
- Speech and swallowing therapy
- Dietary assistance

Early psychological support should be provided for both the patient and their family. Additionally, social services must be available to facilitate social and professional reintegration.

#### Quality assurance

Standardised early rehabilitation assessments or disease-specific scoring systems should be used to evaluate functional deficits. These tools help document treatment outcomes and inform subsequent therapy goals.

### Diagnostic equipment

Rehabilitation facilities must have the necessary equipment for specialised diagnostic procedures, including:

- Ultrasound
- Electrocardiography (ECG)
- Spirometry

### Therapeutic equipment

To support multimodal rehabilitation effectively, facilities must provide equipment that addresses the specific physical limitations of patients.

## Financial considerations

The reimbursement of rehabilitation services varies across European healthcare systems. In most countries, costs are partially covered by basic state funding, with patients often relying on private supplementary insurance for comprehensive coverage. Gaps in state funding can significantly hinder access to high-quality rehabilitative care, particularly for severely injured individuals.

## Conclusion and needs for the future

Rehabilitation must be a cornerstone of holistic trauma care across Europe, ensuring severely injured patients receive tailored, high-quality therapy that addresses both physical and psychological needs. Comprehensive funding frameworks are essential to bridge disparities in access and ensure equitable care. Future efforts should focus on standardizing guidelines, fostering collaboration between rehabilitation facilities, and addressing financial inequalities across European healthcare systems. By integrating these priorities, rehabilitation can significantly enhance the recovery and reintegration of polytrauma patients.

## Bibliography

- Deutsche Gesellschaft für Unfallchirurgie e.V. Weißbuch Schwerverletztenversorgung, 2. Erweiterte Auflage, Supplement 1, Juni 2012.
- Bundesarbeitsgemeinschaft für Rehabilitation e.V. (BAR). Ambulante und stationäre Rehabilitation, Rahmenempfehlungen - Allgemeiner Teil, 1. März 2021. Available from: https://www.bar-frankfurt.de/fileadmin/dateiliste/_publikationen/reha_vereinbarungen/pdfs/MedRehaAllgemein.web.pdf
- Ausbildungs- und Prüfungsverordnung für Masseure und medizinische Bademeister (Artikel 1 der Verordnung über die Ausbildung und Prüfung von Masseuren und medizinischen Bademeistern und zur Änderung verschiedener Ausbildungs- und Prüfungsverordnungen betreffend andere Heilberufe) (MB-APrV). Available from: https://www.bar-frankfurt.de/fileadmin/dateiliste/_publikationen/reha_vereinbarungen/pdfs/MedRehaAllgemein.web.pdf
- Deutsche Gesetzliche Unfallversicherung (DGUV). DGUV Rundschreiben 0238/2019 vom 01.07.2019. Leitfaden zur Neuorganisation des Vergütungssystems in den BG-Einrichtungen (Version 1.6, Stand 17.06.2019).
- Critchfield E, et al. A model of care for community reintegration: the Polytrauma Transitional Rehabilitation Program. Phys Med Rehabil Clin N Am. 2019;30(1):43–54.
- Khan F, Amatya B, Hoffman K. Systematic review of multidisciplinary rehabilitation in patients with multiple trauma. Br J Surg. 2012;99(Suppl 1):88–96.
- NICE guidelines. Available from: https://www.nice.org.uk/guidance/ng211

## Data Availability

No datasets were generated or analysed during the current study.

